# Safety and efficacy of pegunigalsidase alfa in patients with Fabry disease who were previously treated with agalsidase alfa: results from BRIDGE, a phase 3 open-label study

**DOI:** 10.1186/s13023-023-02937-6

**Published:** 2023-10-21

**Authors:** Aleš Linhart, Gabriela Dostálová, Kathy Nicholls, Michael L. West, Camilla Tøndel, Ana Jovanovic, Pilar Giraldo, Bojan Vujkovac, Tarekegn Geberhiwot, Einat Brill-Almon, Sari Alon, Raul Chertkoff, Rossana Rocco, Derralynn Hughes

**Affiliations:** 1 2nd Department of Internal Cardiovascular Medicine, First Faculty of Medicine, Charles University and General University Hospital in Prague, U Nemocnice 2, 128 08 Prague 2, Czech Republic; 2 Department of Nephrology, Royal Melbourne Hospital and The University of Melbourne, Parkville, Australia; 3 Division of Nephrology, Department of Medicine, Dalhousie University, Halifax, NS Canada; 4 Department of Clinical Science, University of Bergen, Bergen, Norway; 5 Nephrology and Rheumatology Unit, Department of Pediatrics, Haukeland University Hospital, Bergen, Norway; 6 Department of Inherited Metabolic Disease, Salford Royal, Salford, England UK; 7 Centro de Investigación Biomédica en Red de Enfermedades Raras, Hospital de Dia Quiron, Zaragoza, Spain; 8 Department of Internal Medicine, General Hospital Slovenj Gradec, Slovenj Gradec, Slovenia; 9 Department of Diabetes, Endocrinology and Metabolism, University Hospitals Birmingham NHS Foundation Trust and University of Birmingham, Birmingham, England UK; 10 Protalix Biotherapeutics, Carmiel, Israel; 11 Chiesi Farmaceutici S.p.A., Parma, Italy; 12 Lysosomal Storage Disorders Unit, Royal Free London NHS Foundation Trust and University College London, London, England UK

## Abstract

**Background:**

Pegunigalsidase alfa is a novel, PEGylated α-galactosidase-A enzyme-replacement therapy approved in the EU and US to treat patients with Fabry disease (FD).

**Objective/methods:**

BRIDGE is a phase 3 open-label, switch-over study designed to assess safety and efficacy of 12 months of pegunigalsidase alfa (1 mg/kg every 2 weeks) treatment in adults with FD who had been previously treated with agalsidase alfa (0.2 mg/kg every 2 weeks) for ≥ 2 years.

**Results:**

Twenty-seven patients were screened; 22 met eligibility criteria; and 20 (13 men, 7 women) completed the study. Pegunigalsidase alfa was well-tolerated, with 97% of treatment-emergent adverse events (TEAEs) being of mild or moderate severity. The incidence of treatment-related TEAEs was low, with 2 (9%) discontinuations due to TEAEs. Five patients (23%) reported infusion-related reactions. Overall mean (SD; n = 22) baseline estimated glomerular filtration rate (eGFR) was 82.5 (23.4) mL/min/1.73 m^2^ and plasma lyso-Gb_3_ level was 38.3 (41.2) nmol/L (men: 49.7 [45.8] nmol/L; women: 13.8 [6.1] nmol/L). Before switching to pegunigalsidase alfa, mean (standard error [SE]) annualized eGFR slope was − 5.90 (1.34) mL/min/1.73 m^2^/year; 12 months post-switch, the mean eGFR slope was − 1.19 (1.77) mL/min/1.73 m^2^/year; and mean plasma lyso-Gb_3_ reduced by 31%. Seven (35%) out of 20 patients were positive for pegunigalsidase alfa antidrug antibodies (ADAs) at ≥ 1 study timepoint, two of whom had pre-existing ADAs at baseline. Mean (SE) changes in eGFR slope for ADA-positive and ADA-negative patients were + 5.47 (3.03) and + 4.29 (3.15) mL/min/1.73 m^2^/year, respectively, suggesting no negative impact of anti-pegunigalsidase alfa ADAs on eGFR slope.

**Conclusion:**

Pegunigalsidase alfa may offer a safe and effective treatment option for patients with FD, including those previously treated with agalsidase alfa.

*TRN*: NCT03018730. Date of registration: January 2017.

**Supplementary Information:**

The online version contains supplementary material available at 10.1186/s13023-023-02937-6.

## Introduction

Fabry disease (FD; Online Mendelian Inheritance in Man® MIM Number: 301500) is a rare, X-linked, lysosomal storage disorder arising from α-galactosidase-A (*GLA*, MIM: 300644) gene mutations, causing deficiency of the α-galactosidase-A (α-Gal-A) enzyme [1, 2]. This deficiency causes progressive accumulation of globotriaosylceramide (Gb_3_) and its deacylated derivative globotriaosylsphingosine (lyso-Gb_3_) in most cell types and body fluids, damaging multiple systems, including cardiovascular, renal, and nervous [2, 3].

In classically affected men, FD symptoms may manifest during childhood and include gastrointestinal complaints, neuropathic pain, hypohidrosis, autonomic dysfunction, and angiokeratomas [4, 5]. Severe complications, including kidney dysfunction and cardiac and cerebrovascular events, emerge during adulthood, frequently resulting in poor quality of life and higher premature death risk [3, 6, 7]. Men with higher residual α-Gal-A enzyme activity generally exhibit milder, later-onset, disease with predominantly single-organ involvement [4, 8]. Women with FD are usually heterozygous, with disease manifestations ranging from asymptomatic to severe, influenced by skewed X-chromosome inactivation patterns [4, 9]. FD renal pathology is associated with progressive chronic kidney disease (CKD) with proteinuria and reduced glomerular filtration rate (GFR), when untreated, progresses to end-stage kidney disease [4, 10]. Renal complications contribute significantly to FD morbidity and mortality [4].

Enzyme-replacement therapies (ERTs) commercially available for FD, agalsidase alfa and agalsidase beta, reduce Gb_3_ accumulation, slow decline in estimated GFR (eGFR), improve cardiovascular symptoms, reduce pain, and improve patient quality of life [11–17]. Despite treatment advances with ERTs, limitations remain. Some patients show limited improvement with FD treatment [18, 19]. Frequent development of antidrug antibodies (ADAs) with neutralizing capabilities can interfere with clinical efficacy [12, 20, 21]. Furthermore, infusion-related reactions (IRRs) associated with ERTs could affect treatment compliance [18, 22–24].

Pegunigalsidase alfa is a chemically modified recombinant human α-Gal-A ERT approved by the European Medicines Agency and US Food and Drug Administration for FD treatment [25]. Pegunigalsidase alfa is modified with polyethylene glycol (PEG), producing PEGylated protein subunits cross-linked into homodimers. As a result of PEGylation, pegunigalsidase alfa has a plasma half-life of about ~ 80 h compared to ~  ≤ 2 h for other currently available ERTs, and carries the theoretical potential for reduced immunogenicity due to epitope masking as suggested by in vitro studies [26]. In ERT-naïve patients, pegunigalsidase alfa treatment resulted in decreased Gb3 kidney deposition, decreased plasma lyso-Gb3, and reduced decline of kidney function with benefits sustained for up to 6 years of follow-up [27, 28].

The objective of BRIDGE was to evaluate the safety and efficacy of switching from agalsidase alfa to pegunigalsidase alfa. Both the pre-switch agalsidase alfa and the post-switch pegunigalsidase alfa treatments were administered per each product’s approved dosage of 0.2 mg/kg and 1 mg/kg, respectively [25, 29].

## Methods

### Study design

BRIDGE (NCT03018730) was a phase 3 open-label, switch-over study designed to assess safety and efficacy of pegunigalsidase alfa (1 mg/kg) administered every 2 weeks (E2W) to adults with FD previously receiving agalsidase alfa (0.2 mg/kg E2W) for ≥ 2 years. During a 3-month screening/pre-switch period, patients were evaluated monthly while continuing their previous regimen of agalsidase alfa; eligible patients then switched to 1 mg/kg pegunigalsidase alfa intravenous (IV) infusion E2W for 12 months. Patients completing the 12-month treatment period were invited to continue treatment in an open-label extension study  (NCT03566017).

### Patients

Adults aged 18–60 years with a documented FD diagnosis and ≥ 1 FD characteristic feature (neuropathic pain, cornea verticillata, and/or clustered angiokeratoma) were eligible. In men, FD was defined by plasma and/or leukocyte α-galactosidase activity less than the lower limit of normal. FD in women was confirmed by historical genetic test based on known pathogenic *GLA* mutations or novel mutations confirmed through a first-degree male relative with FD (Additional file 1: Table S1).

Eligible patients must have received ≥ 2 years of treatment with agalsidase alfa, remaining on a stable dose of > 80% of the labelled dose (0.2 mg/kg) for ≥ 6 months. Additional inclusion criteria were eGFR based on Chronic Kidney Disease-Epidemiology Collaboration equation (eGFR_CKD-EPI_) ≥ 40 mL/min/1.73 m^2^ and ≥ 2 serum creatinine (sCr) evaluations since agalsidase alfa initiation, evaluated within 2 years before screening. Key exclusion criteria included history of type 1 hypersensitivity reaction to agalsidase alfa, renal dialysis or transplant, and acute kidney injury within 12 months before screening. Patients with a urine protein-to-creatinine ratio > 0.5 g/g not treated with an angiotensin-converting enzyme inhibitor (ACEi) or angiotensin receptor blocker (ARB) were excluded, and ACEi or ARB dose changes were not permitted within 4 weeks before screening. See online additional material for all inclusion/exclusion criteria.

### Treatment

Pegunigalsidase alfa infusions were administered at 1 mg/kg E2W, at a volume adjusted according to patient weight, as defined by the protocol. Infusions were initially administered over 3 h (0.83 mL/min), and if tolerated well, infusion time was gradually reduced to 1.5 h with investigator and medical monitor approval. Initial infusions were administered at the study center; subsequent infusions could take place in a home care setting, in accordance with local regulations. Patients were observed for 2 h post-dose, with an option to reduce observation time to 1 h if tolerability was good. Patients previously using medication to prevent infusion reactions with agalsidase alfa continued that medication for initial infusions of pegunigalsidase alfa, then gradually tapered off over the first 2 months of pegunigalsidase alfa treatment, with re-introduction as needed.

### Safety endpoints and assessments

The primary objective was to assess the safety of pegunigalsidase alfa. Treatment-emergent adverse events (TEAEs), clinical laboratory measurements, physical examination results, electrocardiographic findings, injection-site reactions, and IRRs (TEAEs occurring during or within 2 h after the infusion and reported as definitely, probably, or possibly treatment-related) were assessed.

Presence and titers of anti-pegunigalsidase alfa immunoglobulin G (IgG) antibodies were determined using a validated, direct enzyme-linked immunosorbent assay and in vitro enzymatic activity for neutralizing antibodies. Assays were developed and validated according to US Food and Drug Administration (FDA) and European Medicines Agency (EMA) immunogenicity guidelines and performed at a central laboratory (Protalix Biotherapeutics, Carmiel, Israel, a Good Laboratory Practices [GLP]-accredited facility), in accordance with GLP.

### Efficacy endpoints and assessments

The secondary objective was to evaluate efficacy of pegunigalsidase alfa by analyzing plasma lyso-Gb_3_ levels and plasma Gb_3_ levels which represent sensitive biomarkers for FD severity [30–33]. Lyso-Gb_3_, identified as the most sensitive biomarker with clinical applicability, was analyzed in plasma using lipid extraction followed by ultra-performance liquid chromatography/tandem mass spectrometry (UPLC-MS/MS) at a central laboratory (Division of Medical Genetics, Université de Sherbrooke, Québec, Canada). Plasma Gb_3_ was also analyzed using an HPLC system with ESI–MS/MS. Blood samples were collected at the initial screening visit, and after 3, 6, 9, and 12 months of pegunigalsidase alfa treatment.

eGFR was calculated from the CKD-EPI equation, accounting for sCr concentration, age, and sex [34, 35]. Pre-switch eGFR slope calculation included at least 4 sCr values: all available sCr values (≥ 2 values required) within 2 years before screening and during the 3-month screening period, along with Visit 1 pre-infusion sCr (baseline) level. Post-switch eGFR slope calculation included Visit 1 pre-infusion sCr value and measurements every 4 weeks during treatment. Historical sCr values were collected from patient records, and sCr samples collected during the study were analyzed centrally using a substrate-triggered, rate-blanked method utilizing a modification of the Jaffe reaction (Covance Central Laboratory Services Limited Partnership), which is standardized to the isotope dilution mass spectrometry method used to establish the CKD-EPI equation. The assay used in this study results in a colorimetric reaction, where the rate of color formation is proportional to the concentration of creatinine present and is measured photometrically.

To further assess disease-state stability before and after switching to pegunigalsidase alfa, baseline kidney disease status was classified using pre-switch eGFR slopes, based on the 2018 European consensus statement on FD therapeutic goals [4]: stable (eGFR slope ≥  − 3 mL/min/1.73 m^2^/year), progressing (≥ − 5 to <  − 3 mL/min/1.73 m^2^/year), or fast-progressing (< − 5 mL/min/1.73 m^2^/year), and compared to post-switch disease status.

Left ventricular mass (LVM) was measured by standardized cardiac magnetic resonance imaging protocol, images were analyzed centrally, and LVM index (LVMi) was calculated based on body surface area.

### Statistical analysis

Safety population included participants receiving any dose of pegunigalsidase alfa. Efficacy population included patients with ≥ 1 efficacy evaluation after receiving ≥ 1 pegunigalsidase alfa infusion. Efficacy and safety parameters were summarized using descriptive statistics for continuous and categorical variables. Due to limited numbers of patients with FD available for clinical trials, sample size calculation was not performed. Screening/pre-switch and post-switch eGFR slopes were compared with a paired t-test. LVMi change from baseline to month 12 was analyzed post-hoc via one-sample t-test.

## Results

### Patients

Twenty-seven patients from 10 sites were assessed for eligibility, with 22 patients (15 men, 7 women) enrolled and treated from 9 sites (Additional file 1: Figure S1). The safety population included all 22 patients, and the efficacy population included 20 patients (2 patients discontinued due to adverse events [AEs] during the first infusion before efficacy evaluations and were excluded; see below, Safety).

The mean (standard deviation [SD]) age of initial FD therapy was 34.8 (11.9) years. Mean (SD) plasma lyso-Gb_3_ concentration in the safety population was high at 38.3 (41.2) nmol/L (normal range ≤ 2.4 nmol/L; median [range] was 27.6 [1.2–189.4] nmol/L). Mean (SD) baseline plasma lyso-Gb_3_ levels were 49.7 (45.8) nmol/L in men and 13.8 (6.1) nmol/L in women. Sex-based differences in baseline plasma Gb_3_ levels were less pronounced. Additional baseline characteristics are presented in Table 1 for the safety population overall and stratified by gender.

**Table 1:** Patient baseline and demographic characteristics (safety population)

Parameter	Men n = 15	Women n = 7	Overall N = 22
Age (years)			
Mean (SD)	42.7 (10.6)	46.7 (12.3)	44.0 (11.0)
Median (range)	44.0 (24, 60)	50.0 (26, 59)	44.5 (24, 60)
Age starting ERT (years)			
Mean (SD)	32.6 (11.8)	39.4 (11.6)	34.8 (11.9)
Median (range)	37.0 (12, 49)	41.0 (21, 53)	38.0 (12, 53)
% Residual enzyme activity in leukocytes^a^			
Mean (SD)	4.8 (2.5)	27.9 (10.2)	12.2 (12.5)
Median (range)	4.0 (2, 10)	23.7 (16, 46)	5.3 (2, 46)
% Residual enzyme activity in plasma			
Mean (SD)	2.2 (3.2)	28.5 (12.7)	10.6 (14.5)
Median (range)	0.8 (0.1, 13)	23.9 (17, 51)	2.5 (0.1, 51)
Plasma lyso-Gb_3_ (nmol/L)^b^			
Mean (SD)	49.7 (45.8)	13.8 (6.1)	38.3 (41.2)
Median (range)	39.9 (1, 189)	12.9 (7, 23)	27.6 (1, 189)
Plasma Gb_3_ (µmol/L)^c^			
Mean (SD)	6.3 (2.1)	5.5 (1.9)	6.0 (2.0)
Median (range)	6.2 (3, 11)	6.1 (3, 8)	6.2 (3, 11)
eGFR_CKD-EPI_ (mL/min/1.73 m^2^)			
Mean (SD)	80.8 (26.0)	86.1 (17.8)	82.5 (23.4)
Median (range)	78.1 (49, 124)	87.7 (55, 109)	87.0 (49, 124)
Annualized eGFR slope^d^ (mL/min/1.73 m^2^/year)			
Mean (SD)	− 5.4 (7.1)	− 5.0 (4.4)	− 5.3 (6.3)
Median (range)	− 4.4 (− 21, 6)	− 3.7 (− 11, 2)	− 4.3 (− 21, 6)
UPCR categories			
Severely increased, proteinuria (UPCR > 500 mg/g), n (%)	4 (27)	0	4 (18)
Moderately increased (UPCR ≥ 150 mg/g and ≤ 500 mg/g), n (%)	1 (7)	2 (29)	3 (14)
Patients treated with ACEi/ARB, n (%)	8 (53.3)	4 (57.1)	12 (54.5)

*ACEi* angiotensin-converting enzyme inhibitor, *ARB* angiotensin receptor blocker, *eGFR* estimated glomerular filtration rate, *eGFR*_*CKD-EPI*_ eGFR based on the Chronic Kidney Disease-Epidemiology Collaboration equation, *ERT* enzyme replacement therapy, *Gb*_*3*_ globotriaosylceramide, *lyso-Gb*_*3*_ globotriaosylsphingosine, *SD* standard deviation, *UPCR* urine protein-to-creatinine ratio

^a^Percentage of normal laboratory mean

^b^Normal: ≤ 2.4 nmol/L

^c^Normal: ≤ 5 µmol/L

^d^“Historical” eGFR measurements, collected about 2 years prior to study start, were used to calculate the annualized eGFR slope

### Safety

Among 22 patients receiving pegunigalsidase alfa, 21 (96%) reported 127 TEAEs (Table 2). Of these, 114 TEAEs (90% of all TEAEs) in 19 patients were unrelated or unlikely related to treatment. Most (97%) TEAEs were mild or moderate. No deaths were reported. Four patients (all men; 18%) each experienced 1 severe TEAE (also considered serious AEs [SAEs]). Two of these severe TEAEs were type 1 hypersensitivity reactions occurring in 2 patients (9%) during initial study treatment. Both events were definitively treatment-related, resolved within 1 day with the appropriate treatment, and led to study discontinuation. The first hypersensitivity reaction involved nausea, vomiting, itchy eyes, shortness of breath, throat tightness, facial edema, blanching rash, hives, and tachycardia; the other involved, nausea, headache, agitation, edema (hands, periorbital area, tongue), rigor, chills, and decreased blood pressure. Both patients were IgE ADA-positive at baseline pre- and post-infusion assessments (both IgG negative). Other severe TEAEs were infectious mononucleosis (1 patient; 5%) and urinary tract infection (1 patient; 5%), both considered not treatment-related. All other treatment-related TEAEs were mild.

**Table 2:** Overview of TEAEs (safety population)

Parameter	Men n = 15	Women n = 7	Overall N = 22
Patients with ≥ 1 TEAE, n (%)	14 (93)	7 (100)	21 (96)
Patients with ≥ 1 severe TEAE, n (%)	4 (27)	0	4 (18)
Patients with ≥ 1 serious TEAE, n (%)	4 (27)	0	4 (18)
Patients with ≥ 1 TEAE related^a^ to study treatment, n (%)	5 (33)	0	5 (23)
Patients with ≥ 1 TEAE leading to study discontinuation, n (%)	2 (13)	0	2 (9)^b^
Patients with ≥ 1 infusion-related reaction (IRR),^c^ n (%)	5 (33)	0	5 (23)^b^
TEAEs reported in > 2 patients			
Nasopharyngitis, n (%)	5 (33)	2 (29)	7 (32)
Headache, n (%)	3 (20)	2 (29)	5 (23)
Dyspnea, n (%)	2 (13)	1 (14)	3 (14)

*IRR* infusion-related reaction, *TEAE* treatment-emergent adverse event

^a^Possibly, probably, or definitely related

^b^Two patients each experienced one type 1 hypersensitivity infusion-related reaction which led to study discontinuation

^c^TEAEs occurring during infusion or within 2 h after completion of infusion that were reported as possibly, probably, or definitely related to study treatment, excluding injection-site reactions

The most frequently reported TEAEs (> 2 patients) were nasopharyngitis (7 patients; 32%), headache (5 patients; 23%), and dyspnea (3 patients; 14%). All other TEAEs occurred in ≤ 2 patients. In addition to 2 hypersensitivity reaction SAEs leading to discontinuation, 4 TEAEs, each in 1 patient, led to infusion interruption or deferral: transient ischemic attack (considered an FD-related event, in a patient with history of such events), infusion site discomfort, panic attack, and loss of IV access (extravasation or venous ballooning). All 4 events were not treatment-related, and all 4 patients completed the study.

Nine TEAEs (7% of overall TEAEs) in 5 patients (23%), all men, were IRRs. Two of these patients were IgG ADA-positive during the study, and 3 were IgG ADA-negative. Five nonserious IRR TEAEs (itching, redness, and rash) occurred in 1 man (baseline ADA-positive); all resolved. Nonserious nasal congestion occurred in another man (baseline ADA-negative, positive from week 8 onward), was treated, and resolved. Nonserious dizziness occurred in an ADA-negative man and resolved without sequelae. The 2 previously mentioned type 1 hypersensitivity reactions were in IgG ADA-negative patients.

Most laboratory hematology, biochemistry, and urinalysis parameters, along with vital signs and ECG parameters, remained within normal levels, with no notable changes from baseline.

### Immunogenicity

In the efficacy population, 13 patients (65%) were IgG anti-pegunigalsidase alfa ADA-negative at all timepoints, and 7 patients (35%) were ADA-positive at ≥ 1 timepoint. Of the 7 patients who were ADA-positive during the study, 5 (25% of efficacy population; 3 men, 2 women) were ADA-negative at baseline and developed IgG anti-pegunigalsidase alfa responses (induced responses). Of the 5 patients with induced ADA responses: 3 had transient responses and returned to ADA-negative during the study; 2 remained positive through Month 12 (persistently positive). Two additional persistently positive patients (both men) had pre-existing IgG antibodies (Additional file 1: Table S2). These patients completed all study infusions, showed increased titers following pegunigalsidase alfa treatment (titer-boosted ADA response); and only ADAs in these patients demonstrated in vitro enzyme activity neutralization at most timepoints. There was no notable association between IgG ADA positivity and TEAEs. Predosing, IgE positivity to pegunigalsidase alfa was found in 2 patients experiencing hypersensitivity reactions.

### Efficacy

**Fig. 1:**
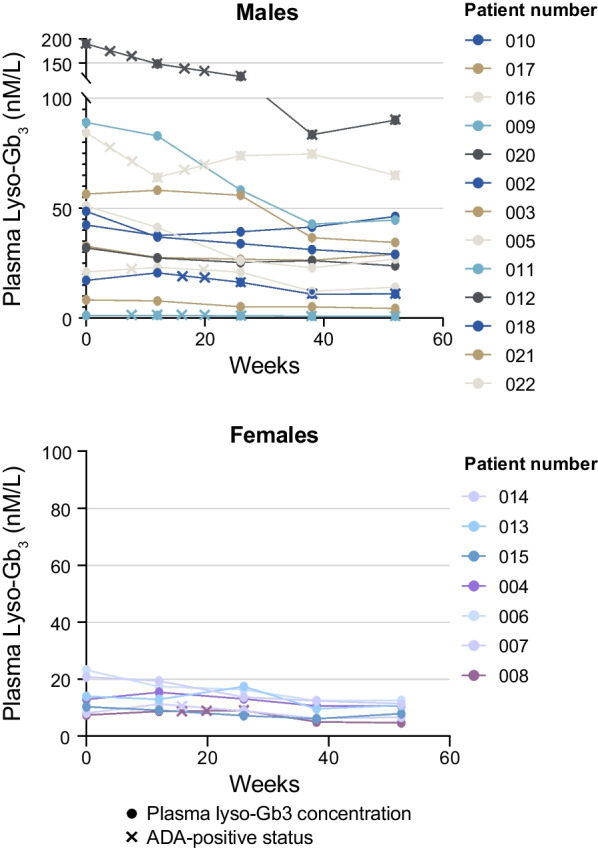
Change in plasma lyso-Gb_3_ in males and females (efficacy population). Anti-pegunigalsidase alfa ADA assessment was conducted at baseline, Weeks 4, 8, 12, 16, 20, 26, 38, and 52; positive status is shown with an x. ADA, antidrug antibody

In the efficacy population, for most patients, plasma lyso-Gb_3_ concentration continuously reduced over the first 9 months and was maintained through Month 12 (Fig. 1). Mean plasma lyso-Gb_3_ concentration was 38.5 nmol/L at baseline and 24.2 nmol/L at Month 12, with mean change of − 14.3 nmol/L (− 31%) in the overall efficacy population (Fig. 1, Table 3). As expected in FD, lyso-Gb_3_ concentrations and absolute observed decreases were higher in men versus women, although relative changes were similar between men and women (32% vs 30% reduction, respectively, Table 3).

**Table 3:** Change in plasma lyso-Gb_3_ and Gb_3_ levels (efficacy population)

Parameter	Men n = 13				Women n = 7				Overall N = 20			
	Baseline	Month 12	Change from baseline	% change	Baseline	Month 12	Change from baseline	% change	Baseline	Month 12	Change from baseline	% change
Plasma lyso-Gb_3_ level, nmol/L												
Mean (SD)^a^	51.8 (49.0)	32.3 (24.9)	− 19.6 (27.2)	− 32.4 (4.8)	13.8 (6.1)	9.2 (2.9)	− 4.6 (3.8)	− 29.8 (4.7)	38.5 (43.3)	24.2 (22.8)	− 14.3 (23.0)	− 31.5 (3.5)
Median (range)	42.4 (1, 189)	29.0 (1, 90)	− 8.2 (− 99, 4)	− 36.1 (− 53, 9)	12.9 (7, 23)	10.6 (5, 13)	− 2.7 (− 11, − 1)	− 23.3 (− 46, − 17)	22.1 (1, 189)	13.4 (1, 90)	− 6.6 (− 99, 4)	− 34.5 (− 53, 9)
Plasma Gb_3_ level, μmol/L												
Mean (SD)^a^	6.4 (2.0)	5.6 (1.4)	− 0.8 (1.5)	− 9.0 (5.6)	5.5 (1.9)	4.7 (1.2)	− 0.8 (1.1)	− 11.2 (6.9)	6.1 (2.0)	5.3 (1.4)	− 0.8 (1.3)	− 9.8 (4.3)
Median (range)	6.2 (3, 11)	5.5 (3, 8)	0.04 (− 4, 1)	0.6 (− 40, 19)	6.1 (3, 8)	4.8 (2, 6)	− 0.8 (− 3, 1)	− 18.4 (− 32, 18)	6.2 (3, 11)	5.3 (2, 8)	− 0.7 (− 4, 1)	− 13.7 (− 40, 19)

*Gb*_*3*_ globotriaosylceramide, *lyso-Gb*_*3*_ globotriaosylsphingosine, *SD* standard deviation, *SE* standard error

^a^Percent change is reported as mean (SE)

A 10% reduction in plasma Gb_3_ concentration was observed, from a mean of 6.1 µmol/L at baseline to 5.3 µmol/L at Month 12 (Table 3). Although absolute values were higher in men than in women (as expected), percentage changes from baseline to Month 12 were similar, with mean reductions of 9% in men and 11% in women (Table 3).

**Fig. 2:**
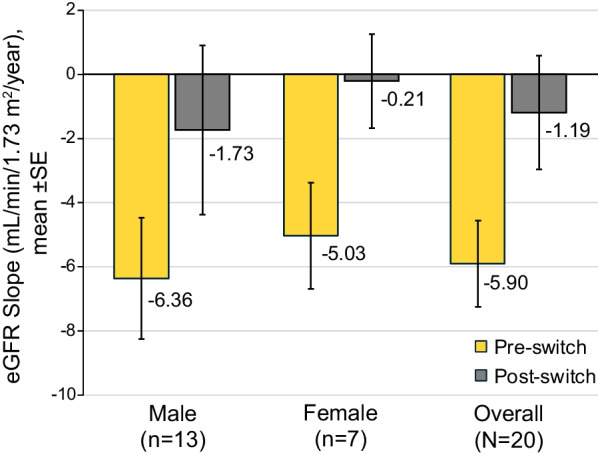
eGFR annualized slope in males, females, and overall study population (efficacy population). *eGFR* estimated glomerular filtration rate, *SE* standard error

In the efficacy population, the mean (SE) annualized “historical” pre-switch eGFR slope, which includes measurements beginning about 2 years before screening through study start and centralized measurements collected during the screening period, was − 5.90 (1.34) mL/min/1.73 m^2^/year (Fig. 2, Additional file 1: Table S3). Mean (SE) post-switch eGFR slope was − 1.19 (1.77), resulting in a mean (SE) change from pre- to post-switch of + 4.70 (2.26; p = 0.051, paired t-test) mL/min/1.73 m^2^/year (Fig. 2).

The mean (SE) annualized pre-switch eGFR slope in men (n = 13) was − 6.36 (1.89), and in women (n = 7) was − 5.03 (1.65) mL/min/1.73 m^2^/year. Post-switch slopes were − 1.73 (2.64) and − 0.21 (1.47) mL/min/1.73 m^2^/year in men and women, resulting in mean (SE) changes of + 4.63 (3.48) and + 4.83 (1.09) mL/min/1.73 m^2^/year, respectively (Fig. 2). Annualized pre- and post-switch eGFR slopes for each patient are shown in Additional file 1: Figures S2 and S3.

**Fig. 3:**
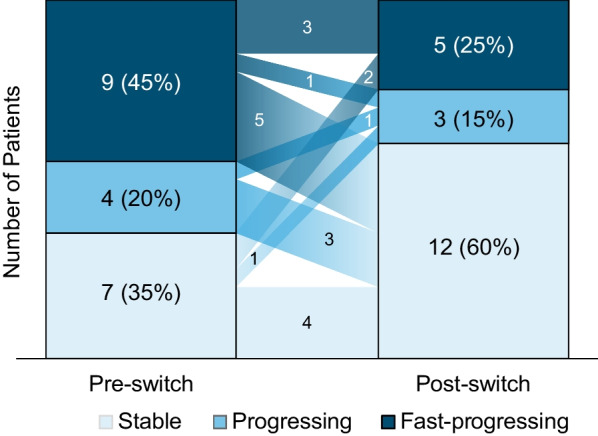
Kidney disease severity status (eGFR slope) shift from pre-switch to post-switch (efficacy population). Fast-progressing: eGFR slope <  − 5 mL/min/1.73 m^2^/year [4]. Progressing: eGFR slope ≥  − 5 to <  − 3 mL/min/1.73 m^2^/year. Stable: eGFR slope ≥  − 3 mL/min/1.73 m^2^/year. *eGFR* estimated glomerular filtration rate

Pre-switch eGFR slopes demonstrated stable disease in 7 (35%) patients and progressing or fast-progressing kidney deterioration in 13 (65%) patients (Fig. 3 and Additional file 1: Table S1 and Figure S2) based on the 2018 European consensus statement on FD therapeutic goals [4]. After 12 months of pegunigalsidase alfa treatment, 9 patients (45%) experienced a positive change in eGFR slope sufficient to move into a different kidney disease group: 1 patient moved from the fast-progressing to progressing category; 5 patients from fast-progressing to stable; and 3 from progressing to stable. Three patients (15%) had negative change resulting in reclassification, and 8 patients (40%) had no change (Additional file 1: Table S1). Across the efficacy population, the number of patients with stable disease increased to 12 (60%), with 8 (40%) demonstrating progressing or fast-progressing kidney disease classification, although 4 of those 8 patients initiated the study in the same category and 1 moved from fast-progressing to progressing post-switch (Fig. 3 and Additional file 1: Table S1 and Figure S2).

Annualized eGFR slopes were similar between ADA-positive and ADA-negative patients pre-switch, at a mean (SE) of − 5.76 (2.85) and − 5.97 (1.48) mL/min/1.73 m^2^/year, respectively. Mean (SE) changes in eGFR slope from pre-switch to post-switch were also similar in ADA-positive (+ 5.47 [3.03] mL/min/1.73 m^2^/year) and ADA-negative (+ 4.29 [3.15] mL/min/1.73 m^2^/year) patients. Of the 2 patients who were ADA-positive at baseline (binding and neutralizing ADAs), 1 patient experienced no change in kidney disease status (eGFR slope − 4.55 pre-switch to − 3.29 mL/min/1.73 m^2^/year post-switch), and 1 experienced a positive change from fast-progressing to stable kidney disease (eGFR slope − 7.49 pre-switch to + 3.59 mL/min/1.73 m^2^/year post-switch).

After 12 months of pegunigalsidase alfa treatment, LVMi in men remained stable, at a mean (standard error [SE]) of 98.3 (7.8) g/m^2^ versus 97.6 (8.9) g/m^2^ at baseline with a mean (SE) change of 2.4 (3.4) (p = 0.50, one-sample t-test; normal range of 57–91 g/m^2^ for men aged 20–60 years [36]); in females, LVMi increased from 66.9 (5.8) g/m^2^ at baseline to 74.1 (7.2) g/m^2^ at 12 months with a mean (SE) change of 7.1 (5.0) but remained within normal range (p = 0.21; 47–77 g/m^2^ for women aged 20–60 years [36]) [37]. LVMi change from baseline between the two sexes was not significantly different (p = 0.45).

## Discussion

Pegunigalsidase alfa is a chemically modified, recombinant human α-Gal-A enzyme with a substantially longer plasma half-life compared with other available ERTs [28, 38, 39]. Safety findings show pegunigalsidase alfa was well-tolerated, with most (97%) TEAEs of mild or moderate severity. Incidence of treatment-related TEAEs (23%), SAEs (18%), and TEAEs causing discontinuation (9%) were low and consistent with previous findings.[28] Five patients (23%) reported mostly mild or moderate IRRs, slightly higher than the approximately 14% reported for agalsidase alfa 0.2 mg/kg E2W, but lower than the 59% infusion-associated reaction rate reported for agalsidase beta 1 mg/kg E2W [29, 40].

Only 2 patients discontinued both due to type 1 hypersensitivity IRRs. Both reactions resolved within 1 day; both patients were positive for IgE anti-pegunigalsidase alfa before receiving their first pegunigalsidase alfa infusion (both negative for IgG ADAs). Across all pegunigalsidase alfa clinical development trials, 4 (3%) pegunigalsidase alfa-treated patients (1 ERT-naive and 3 ERT-experienced patients) experienced anaphylaxis; all during the initial infusion, and all were positive for anti-pegunigalsidase alfa IgE antibodies [25]. Hypersensitivity reactions, including anaphylactic reactions, have been reported in patients receiving other ERTs including agalsidase beta [40]. While the exact mechanism triggering IgE-associated hypersensitivity reactions with ERTs and any associated risk factors are not well understood, it is important that patients are initially exposed to treatment at a treatment center with appropriate medical support measures readily available. In a case of severe hypersensitivity, discontinue the infusion and treat accordingly.

Most patients demonstrated positive change in annualized eGFR slope 12 months after switching to pegunigalsidase alfa. A majority showed significant functional deterioration before enrollment: based on a mean of about 2 years of evaluations before baseline, 65% had eGFR slopes <  − 3 mL/min/1.73 m^2^/year and 45% had fast-progressing kidney disease (eGFR slope <  − 5 mL/min/1.73 m^2^/year), according to the European expert consensus statement on therapeutic goals in FD [4]. The mean change in eGFR slope post-switch was + 4.70 mL/min/1.73 m^2^/year, and post-switch mean eGFR slope was − 1.19 mL/min/1.73 m^2^/year. Although a stabilization or positive change of eGFR slope was seen in most patients, two males demonstrated an opposite trend, independent of ADA status. It has been previously shown that patients on ERT may progress to end-stage renal disease if their renal involvement (fibrosis or proteinuria) is severe before treatment initiation [41]. Overall, the positive mean change in eGFR slope and the increase in the number of patients with stable disease from pre- to post-switch, along with observed decline in lyso-Gb_3_ suggest that some patients may experience clinically meaningful benefit from switching from agalsidase alfa to pegunigalsidase alfa although a treatment period longer than 12 months would be required to confirm this. It is unclear if such treatment benefit could be attributed to differences in drug structure or difference in dose as reported previously in patients switching between agalsidase alfa (dosed 0.2 mg/kg) and agalsidase beta (dosed 1 mg/kg), or both [42, 43]. There is evidence of better clearance of podocyte inclusions with 1 mg/kg agalsidase beta compared with 0.2 mg/kg of either agalsidase alfa or agalsidase beta [44], but there were no significant differences in clinical outcomes between the two agalsidase preparations when both were administered at 0.2 mg/kg [45]. It should also be noted that the phase 3 BALANCE study demonstrated comparable efficacy for change in eGFR slope over 2 years of 1 mg/kg E2W treatment with pegunigalsidase alfa compared with agalsidase beta in adults with deteriorating renal function and long-term history of agalsidase beta treatment [46]. It may therefore be possible the observed effect on renal function in BRIDGE is related to the difference in dose instead of only the unique properties of the therapeutics, but the study was not designed to directly test this.

ERT reduces plasma Gb_3_ and lyso-Gb_3_ concentrations with a rapid (within 3 months) decrease observed in ERT-naïve men with FD, that is generally sustained over 12 months and can extend up to 60 months [13, 47, 48]. In BRIDGE, patients switching to pegunigalsidase alfa showed further reduction in plasma lyso-Gb_3_ of − 14.3 nmol/L (− 31%) over 12 months, suggesting that switching from agalsidase alfa to pegunigalsidase alfa could further improve this disease marker. This change was sustained over 12 months of pegunigalsidase alfa treatment in all patients, consistent with previous findings [28].

Seven of the 20 patients in the efficacy population were positive for IgG anti-pegunigalsidase alfa ADAs at ≥ 1 timepoint during the study. Two of these patients, both men, had pre-existing ADAs at baseline with neutralizing activity and remained ADA-positive during pegunigalsidase alfa treatment. The pre-existing ADAs against pegunigalsidase alfa occurred due to cross-reactivity to the enzyme components of the amino acid sequence shared between pegunigalsidase alfa and agalsidase alfa, as agalsidase alfa is not PEGylated nor has plant glycans [26]. The other 5 developed ADAs transiently (n = 3) or persistently (n = 2), with none having neutralizing enzymatic activity in vitro. In agalsidase beta trials, 83% of patients developed ADAs, and in a registry of > 800 patients with FD treated with agalsidase beta, 73% of males were ADA-positive during treatment [20, 40]. In agalsidase alfa trials, 24–56% of men developed ADAs, with a high incidence of neutralizing antibodies, although a number of them were ERT-naïve [12, 20, 40, 49], in contrast to the patients in the study reported here. It should be noted, however, that comparing incidence of ADA positivity across ERT trials is challenging due to differences in seropositivity assessment methods and the ERT doses studied. Dose may impact immunogenicity as demonstrated by a 1 mg/kg agalsidase beta dose resulting in higher α-Gal-A antibody production than a 0.2 mg/kg agalsidase alfa dose [50, 51]. However, considering the higher incidence of ADAs observed with 1 mg/kg of agalsidase beta than 1 mg/kg pegunigalsidase alfa, and the absence of treatment-induced antibodies among patients receiving 2 mg/kg of pegunigalsidase alfa, other factors such as manufacturing and design of the products may influence immunogenicity [28, 52, 53]. While it may be possible that the observed effect in BRIDGE is dose-related, this study aimed to assess only the approved dosages. Further studies are needed to clarify the potency and immunogenicity of the currently available ERTs. Neutralizing ADAs have been associated with significantly worse clinical outcomes in FD [12, 20, 21]. Neutralizing activity against pegunigalsidase alfa was detected in vitro only in the 2 patients who were IgG-positive at study baseline. In both patients, baseline plasma lyso-Gb_3_ concentrations were initially high and declined consistently in response to pegunigalsidase alfa treatment. Furthermore, 1 of these patients experienced improvement from fast-progressing to stable kidney disease. These results suggest that in the presence of cross-reacting neutralizing antibodies against pegunigalsidase alfa, treatment efficacy could be maintained at least in the context of switching from a lower dose ERT. In this study, anti-pegunigalsidase alfa ADAs had no apparent negative impact on eGFR slope although a longer follow up would be needed. Routine monitoring of ADA status in patients receiving ERT is important particularly in males with a classic FD phenotype who are at highest risk of developing ADAs and experiencing infusion or immune reactions [25, 54].

The findings from the BRIDGE study indicate that pegunigalsidase alfa is an effective treatment in adults with FD, with a favorable safety profile, consistent with previous phase 1/2 study results [28]. Switching from agalsidase alfa to pegunigalsidase alfa appears tolerable, and in most patients may provide additional clinical benefit, particularly with respect to kidney function trajectory and the FD biomarker plasma lyso-Gb_3_. Most (90%) patients elected to continue treatment in the open-label extension (PB-102-F60), attesting to the tolerability of pegunigalsidase alfa and the treatment benefit perceived by patients and their treating physicians.

This study’s main limitation was the uncontrolled period of agalsidase alfa treatment before the pre-switch study period. During this time, clinical measures were nonstandardized, and sCr measurements from local laboratories were collected from patient files, which contained a small but variable number of pre-enrollment data points (Additional file 1: Figure S3), in contrast to the controlled assessments during the study screening and treatment periods, which used a central laboratory and measured creatinine by enzymatic assay. However, given that the screening/pre-switch period was only 3 months, use of additional historical creatinine values of about 2 years pre-switch allowed for additional observations about the patients’ disease trajectories pre- and post-switch. These observations were made in consideration of the known limitations regarding historical values, and the main study efficacy result was restricted to the rate of eGFR decline observed during pegunigalsidase alfa treatment. An additional limitation was the lack of data on plasma Gb_3_ and lyso-Gb_3_ before initiation of agalsidase alfa, allowing us to compare only reductions achieved by pegunigalsidase alfa treatment to levels that were likely already reduced from previous treatment. Lastly, the trial was not designed to assess whether the efficacy findings are due to the PEGylated structure of pegunigalsidase alfa or to the administration of a higher dose relative to agalsidase alfa as per each product’s approved dosage of 1 mg/kg versus 0.2 mg/kg, respectively.

Although ERTs have successfully treated patients with FD, the limitations that have arisen since their approval highlight the need for new treatment options. Pegunigalsidase alfa may offer an effective treatment option for patients with FD, including those previously treated with agalsidase alfa.

A plain language summary of this study is available as Additional file 2 (Additional Text: Pegunigalsidase alfa treatment for adults with Fabry disease).

### Supplementary Information

**Additional file 1**: All inclusion/exclusion criteria, treatment, and safety/efficacy assay details; a selection of tables and figures.

**Additional file 2**: A plain language summary of this study.

## Data Availability

We will approve or deny data requests from external parties on a case-by-case basis. Chiesi reserves the right to deny requests for all legally appropriate reasons. Data requests that risk sharing participant-level data or proprietary information will not be approved.

## Declarations

### Required declaration

The results presented in this paper have not been published previously in whole or part, except in abstract format.
