# Supplementary material for: Safety and efficacy of pegunigalsidase alfa in patients with Fabry disease who were previously treated with agalsidase alfa: results from BRIDGE, a phase 3 open-label study
Source: Orphanet J Rare Dis. 2023 Oct 21;18:332. doi: 10.1186/s13023-023-02937-6 (PMC10589982; doi:10.1186/s13023-023-02937-6)

**Additional online material for:**

**Safety and efficacy of pegunigalsidase alfa in patients with Fabry disease who were previously treated with agalsidase alfa: results from BRIDGE, a phase 3 open-label study**

**Inclusion criteria**

Patients were required to be aged 18 to 60 years, and those having partners of child-bearing potential were required to agree to use a medically acceptable method of contraception.

**Exclusion criteria**

The presence of any of these criteria led to exclusion from the study:

- History of anaphylaxis
- History of acute kidney injury in the 12 months prior to the pre-switch period, including specific kidney diseases (eg, acute interstitial nephritis, acute glomerular and vasculitic renal diseases), nonspecific conditions (eg, ischemia, toxic injury), and extrarenal pathology (eg, prerenal azotemia, acute postrenal obstructive nephropathy)
- Urine protein-to-creatinine ratio of >0.5 g/g and not treated with an angiotensin-converting enzyme inhibitor or angiotensin receptor blocker
- Known history of hypersensitivity to gadolinium contrast agent not managed by premedication
- Pregnancy, planning to become pregnant, or breastfeeding during the study
- Cardiovascular (myocardial infarction, unstable angina) and/or cerebrovascular (stroke, transient ischemic attack) event ≤6 months before pre-switch period
- Congestive heart failure New York Heart Association Class IV
- Any medical, emotional, behavioral, or psychological condition that, in the judgment of the investigator or the medical monitor, would have interfered with study compliance

**Online additional table S1.** Patient sex, mutation, phenotype (safety population), and kidney disease group

| **Patient Number** | **Sex** | **Mutation^a^** | **Kidney Disease Group** | |
| --- | --- | --- | --- | --- |
|  |  |  | **Pre-Switch** | **Post-Switch** |
| 006 | Female | c.671A>C, p.Asn224Thr | stable | stable |
| 007 | Female | c.671A>C, p.Asn224Thr | stable | stable |
| 016 | Male | c.1212_1214del, p.Arg404del | stable | stable |
| 002 | Male | c.454T>C, p.Tyr152His | stable | stable |
| 009 | Male | c.369+5G>T | stable | progressing |
| 017 | Male | c.644A>G, p.N215S | stable | fast-progressing |
| 020 | Male | c.406G>T, p.D136Y | stable | fast-progressing |
| 014 | Female | c.1229C>T, p.Thr410lle;Nil | progressing | stable |
| 004 | Female | c.427G>C, p.Ala143Pro | progressing | stable |
| 011 | Male | c.674_732del, p.His225Leufs*5 | progressing | stable |
| 012 | Male | c.674_732del, p.His225Leufs*5 | progressing | progressing |
| 013 | Female | c.1074_1075del | fast-progressing | stable |
| 015 | Female | c.800T>G, p.Met267Arg | fast-progressing | progressing |
| 008 | Female | c.511G>C, p.Gly171Arg | fast-progressing | fast-progressing |
| 010 | Male | c.178_719del | fast-progressing | stable |
| 005 | Male | c.801+48T>G | fast progressing | stable |
| 018 | Male | c.793C>T, p.Pro265Ser | fast-progressing | stable |
| 022 | Male | c.269G>A, Cys90Tyr | fast-progressing | stable |
| 003 | Male | c.871G>C, p.Ala291Pro | fast progressing | fast-progressing |
| 021 | Male | c.974G>A | fast-progressing | fast-progressing |
| 001^b^ | Male | c.1181_1192del | -- | -- |
| 019^b^ | Male | c.815A>G, p.Asn272Ser | -- | -- |

^a^cDNA mutation preceded by “c,” amino acid change preceded by “p.”

^b^Discontinued patient, not included in efficacy population.

**Online additional table S2.** Immunogenicity in men versus women subgroups and overall (safety) population

|  | | **Men**  **n=15** | **Women**  **n=7** | **Overall**  **N=22** |
| --- | --- | --- | --- | --- |
| **Presence of IgG** | | | | |
| **Baseline, n (%)** | n | 15 | 7 | 22 |
|  | Positive, n (%) | 2 (13) | 0 | 2 (9) |
|  | Negative | 13 (87) | 7 (100) | 20 (91) |
| **Any post-baseline visit,^a^ n (%)** | n | 15 | 7 | 22 |
|  | Positive, n (%) | 5 (33) | 2 (29) | 7 (32) |
|  | Negative, n (%) | 10 (67) | 5 (71) | 15 (68) |
| **Week 52, n (%)** | n | 13 | 7 | 20 |
|  | Positive | 4 (31) | 0 | 4 (20) |
|  | Negative | 9 (69) | 7 (100) | 16 (80) |
| **Transient/tolerized^b^** | n (%) | 1/5 (20) | 2/2 (100) | 3/7 (43) |
| **Presence of Neutralizing Antibody^c^** | | | | |
| **Baseline** | n | 2 | 0 | 2 |
|  | Positive, n (%) | 2 (100) | 0 | 2 (100) |
|  | Negative, n (%) | 0 | 0 | 0 |
| **Any post-baseline visit^a^** | n | 5 | 2 | 7 |
|  | Positive, n (%) | 2 (40) | 0 | 2(29) |
|  | Negative, n (%) | 3 (60) | 2 (100) | 5 (71) |
| **Week 52** | n | 4 | 0 | 4 |
|  | Positive, n (%) | 2 (50) | 0 | 2 (50) |
|  | Negative, n (%) | 2 (50) | 0 | 2 (50) |

^a^Positive, if positive at any visit; negative, if negative at all visits.

^b^Number of patients who became negative/total number of patients who were ADA positive at any post-baseline visit.

^c^Neutralizing activity was evaluated only in ADA-positive samples.

ADA, antidrug antibody; IgG, immunoglobulin G.

**Online additional table S3.** Change in eGFR slope (efficacy population).

| **eGFR slope (mL/min/1.73 m^2^/year)** | **Men**  **n=13** | | | | **Women**  **n=7** | | | | **Overall**  **N=20** | | | |
| --- | --- | --- | --- | --- | --- | --- | --- | --- | --- | --- | --- | --- |
|  | **Pre-switch** | **Post-switch** | **Change from pre- to post-switch** | **p-value^a^** | **Pre-switch** | **Post-switch** | **Change from pre- to post-switch** | **p-value^a^** | **Pre-switch** | **Post-switch** | **Change from pre- to post-switch** | **p-value^a^** |
| Mean (SE) Median (range) | −6.4 (1.9)  −4.6 (−21, 5) | −1.7 (2.6)  −1.1 (−19, 14) | 4.6 (3.5)  3.2 (−18, 22) | 0.21 | −5.0 (1.7)  −3.7 (−11, 2) | −0.2 (1.5)  1.4 (−6, 4) | 4.8 (1.1)  5.9 (−0.1, 8) | 0.004 | −5.9 (1.3)  −4.4 (−21, 5) | −1.2 (1.8)  −0.7 (−19, 14) | 4.7 (2.3)  5.0 (−18, 22) | 0.051 |

^a^p-values and 95% CIs are based on t-distribution (paired t-test).

eGFR, estimated glomerular filtration rate; SE, standard error.

**Online additional figure S1.** BRIDGE study patient disposition.

AE, adverse event; FD, Fabry disease.

**Online additional figure S2.** Kidney disease severity status (eGFR slope) shift from pre-switch to post-switch (efficacy population).

eGFR, estimated glomerular filtration rate.

**Online additional figure S3.** Pre-switch and post-switch eGFR measurements and annualized slope (efficacy population). Light blue squares indicate pre-switch historical eGFR measurements.

Light blue triangles indicate pre-switch measurements within the study period. Dark blue indicates post-switch measurements.

Pre- and post-switch annualized eGFR slope values are shown.


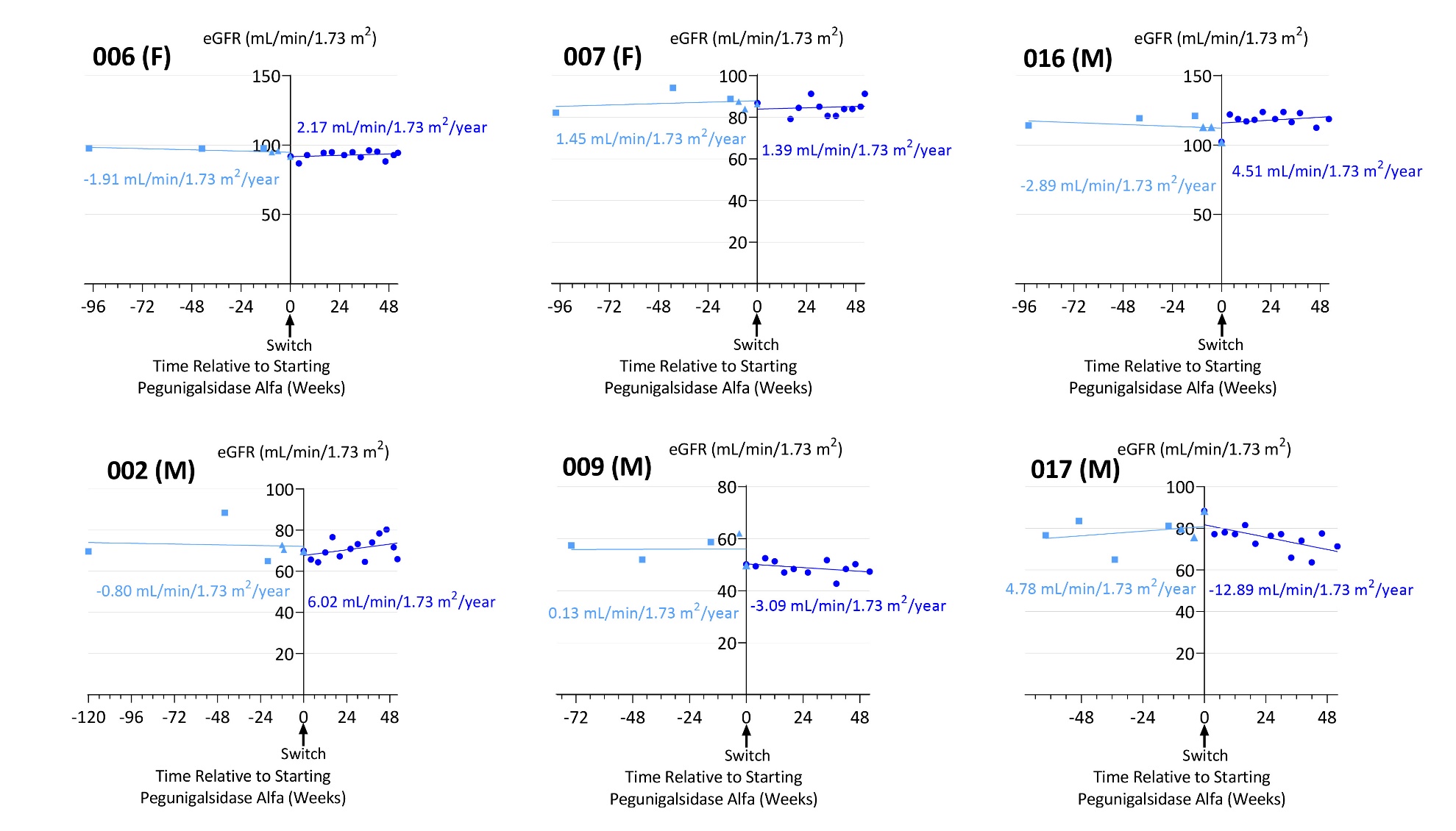


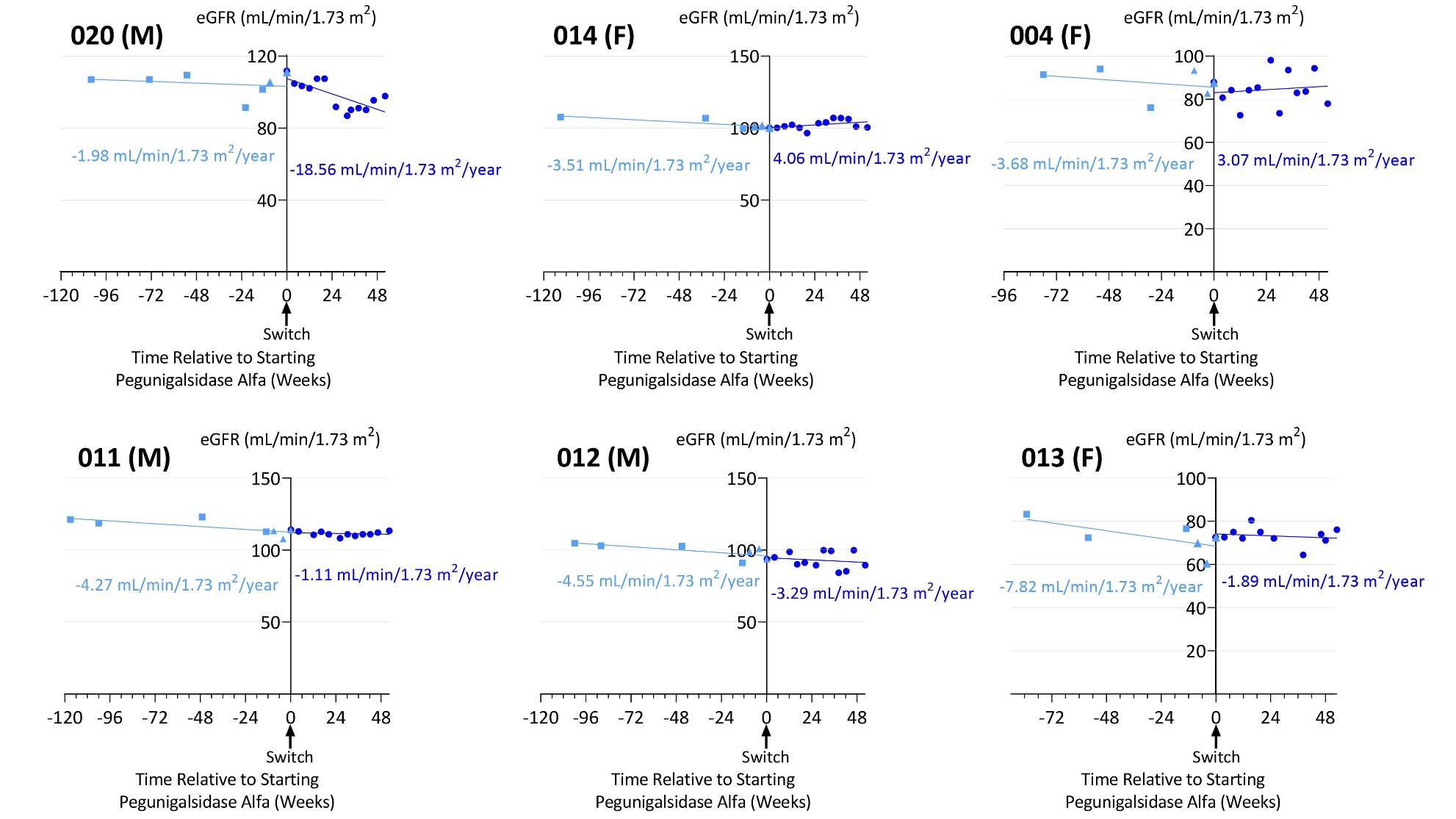


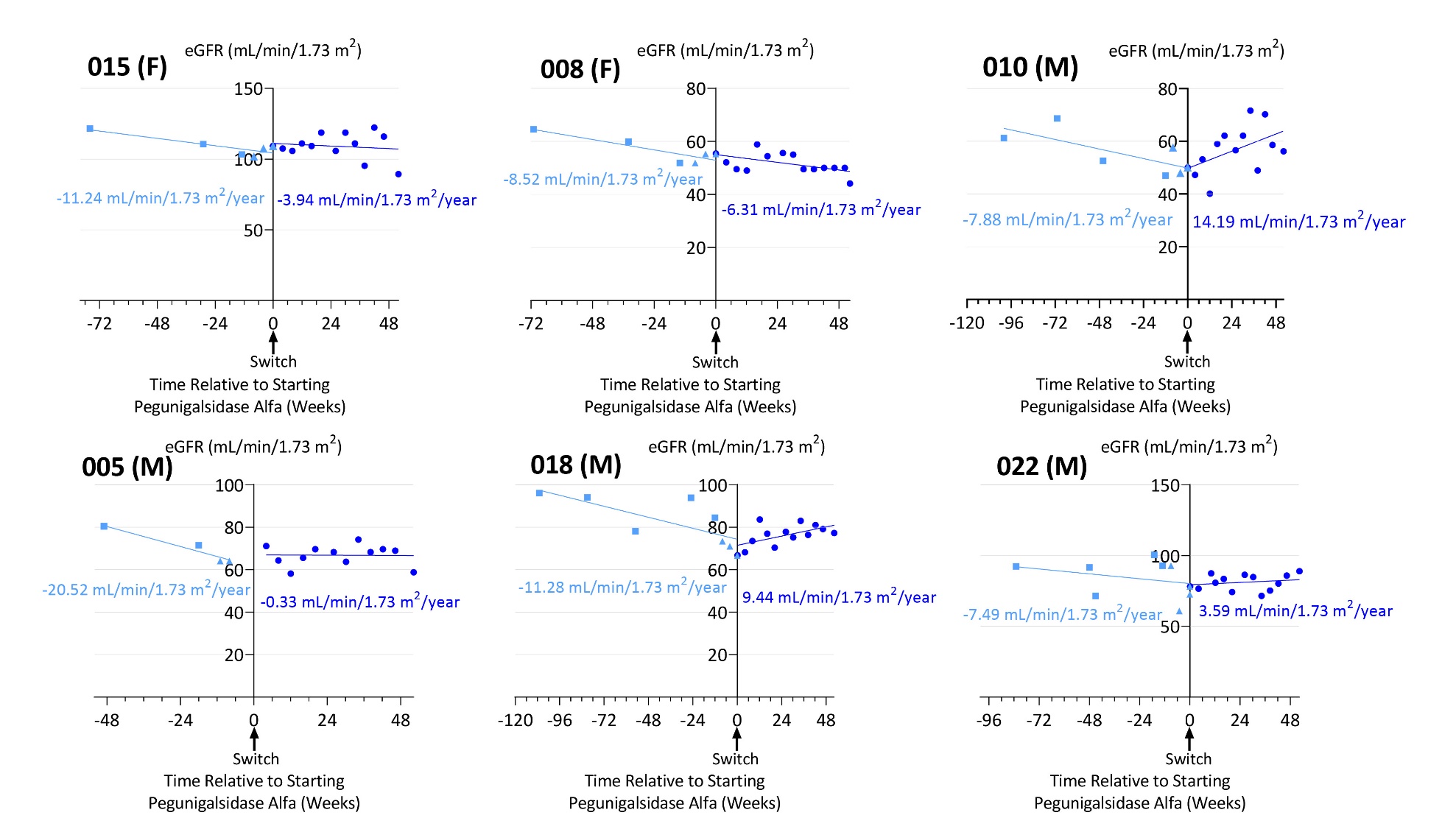


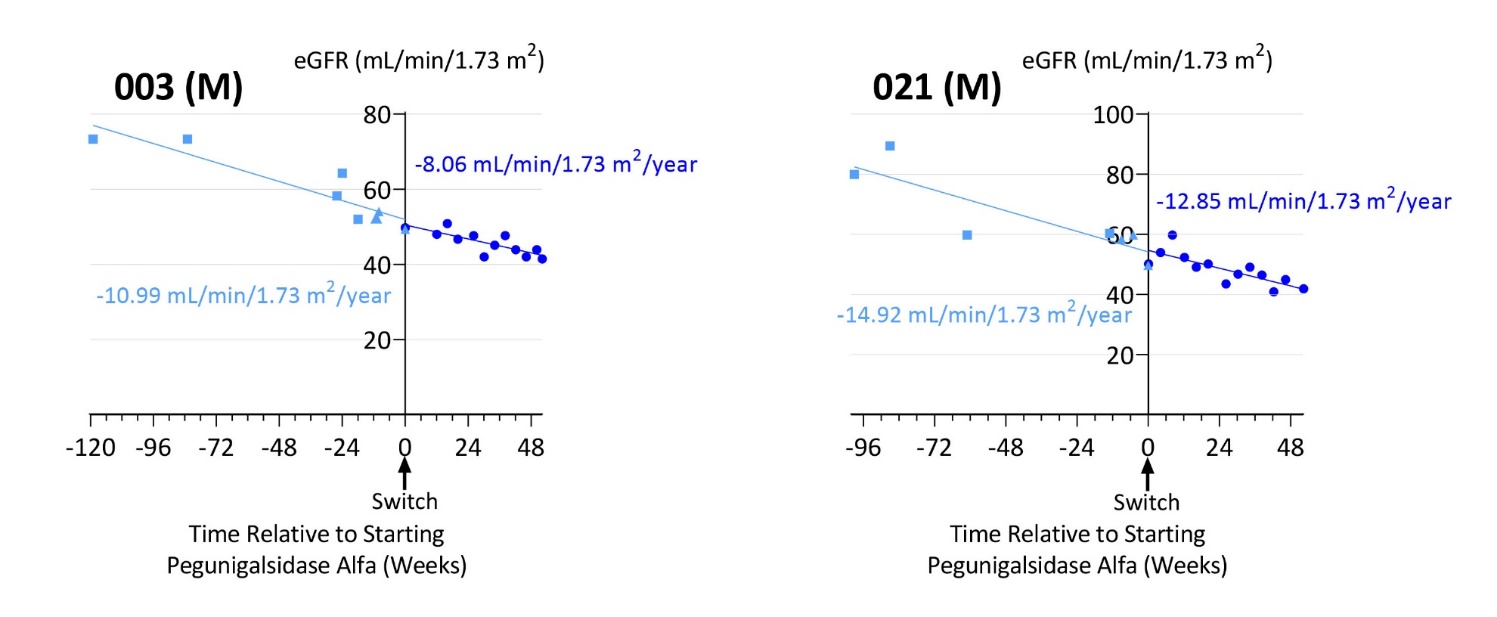

Supplement: Supplementary file 1 — Additional file 1: All inclusion/exclusion criteria, treatment, and safety/efficacy assay details; a selection of tables and figures. [file 13023_2023_2937_MOESM1_ESM.docx]
