# Supplementary material for: Safety and efficacy of pegunigalsidase alfa in patients with Fabry disease who were previously treated with agalsidase alfa: results from BRIDGE, a phase 3 open-label study
Source: Orphanet J Rare Dis. 2023 Oct 21;18:332. doi: 10.1186/s13023-023-02937-6 (PMC10589982; doi:10.1186/s13023-023-02937-6)
Supplement: Supplementary file 2 — Additional file 2: A plain language summary of this study. [file 13023_2023_2937_MOESM2_ESM.pdf]

BRIDGE STUDY

Pegunigalsidase alfa treatment for adults with Fabry disease

Additional online information to Linhart A, et al. *Orphanet Journal of Rare Diseases*. 2023.

This is a plain language summary of an article about the BRIDGE study, which was published in *Orphanet Journal of Rare Diseases* in 2023.

How to say...

Pegunigalsidase alfa: "PEG-oo-nee-gal-suh-days al-fuh"
Agalsidase alfa: "AY-gal-suh-days al-fuh"
Fabry: "FAB-ree"

What is Fabry disease?

Fabry disease is an inherited disorder where an enzyme called alpha galactosidase A, which normally breaks down certain fats, does not work properly. Enzymes are molecules in the body that are part of healthy cell function and can create or break down fats, sugars, or proteins. For people with Fabry disease, the faulty alpha galactosidase A enzyme leads to high levels of certain fats called **Gb_3** and **lyso-Gb_3**. These fats build up in the person's blood and throughout their bodies. This can cause pain and problems with the kidney, heart, and brain. Fabry disease affects both men and women and is a lifelong condition.

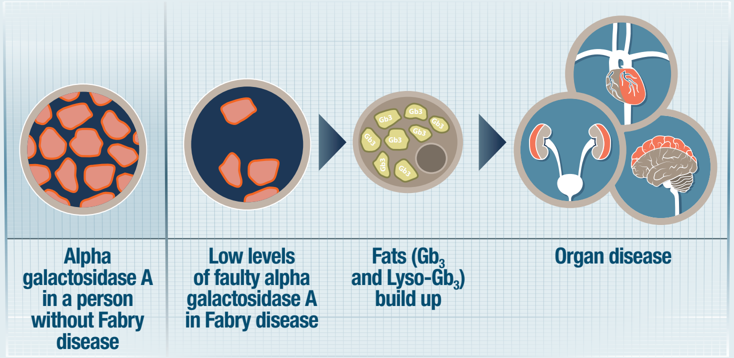

How is Fabry disease treated?

One way to treat Fabry disease is with a type of medication called **enzyme replacement therapy**. This medication replaces the faulty alpha galactosidase A enzyme so people with Fabry disease can break down Gb_3 and lyso-Gb_3. This prevents these fats from building up and protects the kidneys, liver, and heart, so that those organs may continue to work. An enzyme replacement therapy called **agalsidase alfa** is currently available for people with Fabry disease.

Individual people respond to therapies in different ways, so some people still have symptoms of Fabry disease while taking their medication. To try to help with this, another enzyme replacement therapy called **pegunigalsidase alfa** is approved to treat people with Fabry disease.

Pegunigalsidase alfa may last longer in the body than other enzyme replacement therapies, which could help people with Fabry disease control their symptoms better while lowering their levels of Gb_3 and lyso-Gb_3. Agalsidase alfa and pegunigalsidase alfa are each given through a needle into a vein, called an intravenous or an IV infusion.

Why was the study done?

This study was done to test the effects of switching to pegunigalsidase alfa in adults with Fabry disease who were stable on agalsidase alfa.

What questions were answered by the results of this study?

In people with Fabry disease who were switched from treatment with agalsidase alfa to pegunigalsidase alfa, researchers wanted to understand:

- **What were the safety results after switching to treatment with pegunigalsidase alfa?**
- **What effects on the kidney were seen after switching to pegunigalsidase alfa?**
- **What were the effects of switching to pegunigalsidase alfa on Gb_3 and lyso-Gb_3 levels?**

What was the study plan?

At the start of this study, all the participants switched from treatment with agalsidase alfa to pegunigalsidase alfa.

This study included **22 participants** who received study treatment in Australia, Canada, Czechia, the Netherlands, Norway, Slovenia, Spain, and the UK. The participants were between 24 and 60 years old; the average age was 44 years. There were 7 female and 15 male participants in this study.

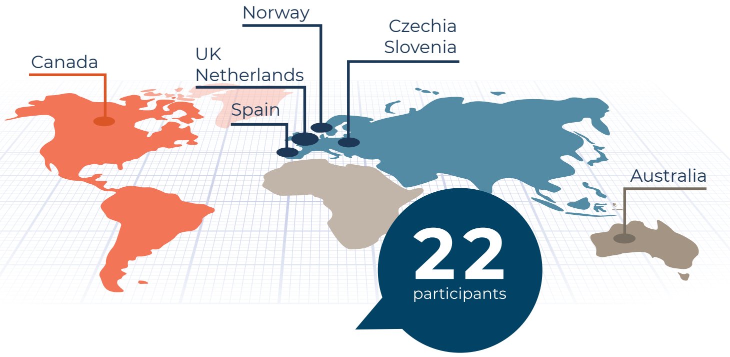

What treatments did the participants receive?

Before the study: Agalsidase alfa

All of the participants received agalsidase alfa for at least 2 years before the start of this study

During the study: Pegunigalsidase alfa

22 participants

Given by an IV infusion once every 2 weeks

The participants received pegunigalsidase alfa for up to 12 months

What did the results of the BRIDGE study show?

What were the safety results of the study?

Overall, pegunigalsidase was well-tolerated by patients participating in the BRIDGE study.

The side effects that participants have during a clinical study, whether they are related to study treatment or not, are registered as **adverse events**. All medications can have adverse events. Adverse events are considered serious when they are life-threatening, cause lasting problems, or result in admission to hospital.

In this study, most (97%) of the adverse events were mild or moderate in severity. The 2 participants who stopped treatment in the study due to adverse events had their symptoms resolve within 1 day of stopping pegunigalsidase alfa treatment.

How many participants had...?	Percent affected and number of participants affected
**Adverse events that happened during the study**	**96% (21)**
Inflammation of the nose and throat	32% (7)
Headache	23% (5)
Infusion site reaction	23% (5)
Difficulty breathing	14% (3)
**Adverse events related to study treatment**	**23% (5)**
**Serious adverse events**	**18% (4)**
**Adverse events that led the participant to stop the study**	**9% (2)**

What effects on the kidney were seen after switching to pegunigalsidase alfa?

The researchers used the results of a blood test to measure how well the participants' kidneys were working.

The researchers counted the number of people with kidneys that were either **stable**, **getting worse**, or **rapidly getting worse**. Stable means the kidneys were not working any worse or better over a certain time period. 9 (45%) of the 22 participants had kidneys that started to work better during the study.

How well the participants kidneys were working before and after receiving pegunigalsidase alfa

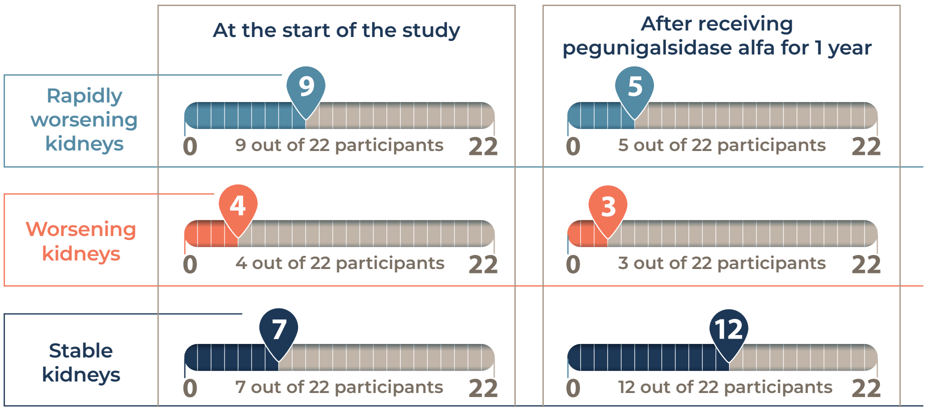

What were the effects of switching to pegunigalsidase alfa on Gb_3 and lyso-Gb_3 levels?

The researchers measured the amount of Gb_3 and lyso-Gb_3 in the participants' bodies using blood and urine tests and found that after switching to pegunigalsidase alfa, they had lower amounts of these fats. They found that, on average, 1 year of pegunigalsidase alfa treatment lowered the participants' Gb_3 levels by **10%** and lyso-Gb_3 by **31%** compared to their levels at the start of the study.

What did this study tell us and why is it important?

Overall, the results from the BRIDGE study showed that pegunigalsidase alfa was well-tolerated. This study also showed that most people who switched from agalsidase alfa to pegunigalsidase alfa had lower levels of Gb_3 and lyso-Gb_3 in their blood and had similar or better kidney function than they did when the study started. This means pegunigalsidase alfa provides a new treatment option for people with Fabry disease, including people who have taken agalsidase alfa in the past.

More information

This summary is based on the article called "Safety and efficacy of pegunigalsidase alfa in patients with Fabry disease who were previously treated with agalsidase alfa: results from BRIDGE, a phase 3 open-label study" which was published in *Orphanet Journal of Rare Diseases* in 2023. You can read more about the BRIDGE study at: https://www.clinicaltrials.gov/ct2/show/NCT03018730.

The BRIDGE study was sponsored by Protalix Ltd. Medical writing support for this summary was provided by Kelsey Hodge-Hanson of Oxford PharmaGenesis, Inc., and was funded by Chiesi. The authors thank all the study participants, their families, and the researchers. Chiesi also thanks Lori Larese Wise, living with Fabry disease, for her editorial review of this summary. The original authors of the full article reviewed and approved this summary.
